# Comparative evaluation of oral versus intravenous antibiotic prophylaxis prior to obstetric and gynecological procedures

**DOI:** 10.6026/973206300223101

**Published:** 2026-05-31

**Authors:** Pradeep Rathore, Ashis Tiwari, Shambhavi Soni

**Affiliations:** 1 Department of General Surgery, Government Medical College, Datia, Madhya Pradesh, India; 2 Department of General Medicine, Government Medical College, Datia, Madhya Pradesh, India; 3 Department of Obstetrics and Gynaecology, Government Medical College, Datia, Madhya Pradesh, India

**Keywords:** Antibiotic prophylaxis, obstetric surgery, gynecological surgery, surgical site infection (SSI), oral antibiotics, intravenous antibiotics

## Abstract

Surgical site infections (SSI) remain among the most common post-operative complications following gynecological and obstetric surgeries. Pre-operative antibiotic prophylaxis plays a key role in preventing SSIs, with intravenous administration serving as the conventional standard. Recent interest has turned toward oral antibiotic prophylaxis as a more convenient and cost-effective alternative. Hence, this prospective comparative study included 221 women undergoing elective gynecological or obstetric procedures, randomly assigned to receive either oral antibiotics (Group A) or intravenous antibiotics (Group B) prior to surgery. Post-operative outcomes, including surgical site infection, febrile morbidity, urinary tract infection duration and hospital stay, were evaluated. Thus, we show the equivalent efficacy between oral and intravenous prophylaxis in infection prevention, advancing clinical practice by supporting oral antibiotic use as a safe, practical and economical alternative in suitable surgical cases.

## Background:

Post-operative morbidity is further exacerbated by surgical site infections (SSI), which continue to rank among the most prevalent healthcare-associated illnesses that may develop after surgical operations. Prolonged hospitalization and increased healthcare costs. In obstetric and gynecological surgeries, infections such as wound infection, pelvic abscess and urinary tract infection can complicate post-operative recovery and adversely affect patient outcomes. Studies estimate that the incidence of SSI in obstetric and gynecological procedures ranges from 3% to 10%, depending on the type of surgery and patient risk factors [1]. Antibiotic prophylaxis is an essential strategy in reducing post-operative infections. To avoid bacterial colonization during surgery, it is important to ensure that tissues have an acceptable concentration of antimicrobials when the incision is made. This is the basic idea of prophylactic antibiotic therapy. To maximize the avoidance of surgical site infection, some professional organizations advise taking prophylactic antibiotics no later than 60 minutes before the incision is made. These organizations include the World Health Organization and the Centers for Disease Control and avoidance [2, 3]. Gynecological surgeries are generally classified as clean-contaminated procedures because the genital tract is entered during surgery. The vaginal flora consists of a variety of aerobic and anaerobic microorganisms including Escherichia coli, Staphylococcus aureus, Streptococcus species and Bacteroides species. These organisms may cause post-operative infections if introduced into surgical wounds during procedures such as hysterectomy, cesarean section, or myomectomy. Therefore, prophylactic antibiotics with broad-spectrum coverage are commonly recommended [4].

Traditionally, intravenous antibiotic prophylaxis has been considered the gold standard because it ensures rapid systemic absorption and adequate tissue levels during surgery. Intravenous administration of cephalosporins such as cefazolin or cefuroxime is commonly practiced in obstetric and gynecological procedures. However, intravenous antibiotics require hospital resources, trained personnel and intravenous access, which may not always be readily available in resource-limited settings [5, 6]. Recently, interest has grown in evaluating the use of oral antibiotics as an alternative method of prophylaxis. Oral antibiotics have several advantages including ease of administration, lower cost and reduced need for intravenous access. Moreover, oral prophylaxis may be particularly useful in outpatient or minimally invasive procedures where hospitalization is brief [7]. Oral antibiotic prophylaxis may be an effective substitute for intravenous administration, according to a recent randomized clinical trial that compared the two methods for preventing surgical site infections in gynecological and obstetric procedures. The two groups had similar infection rates [8, 9]. Therefore, it is of interest to compare oral and intravenous antibiotic prophylaxis among women undergoing obstetric and gynecological interventions.

## Materials and Methodology:

## Study design:

The researchers at this tertiary care teaching hospital's obstetrics and gynecology department set out to perform a prospective comparison study.

## Study duration:

The study was conducted over a period of 18 months.

## Study population:

Women undergoing elective obstetric or gynecological surgical procedures such as:

[1] Cesarean section

[2] Abdominal hysterectomy

[3] Vaginal hysterectomy

[4] Ovarian cystectomies were included in the study.

## Sample size:

A total of 220 patients were included in the study and randomly allocated into two equal groups:

[1] Group A (n=110): Oral antibiotic prophylaxis

[2] Group B (n=110): Intravenous antibiotic prophylaxis

## Inclusion criteria:

[1] Women aged 18-50 years

[2] Patients undergoing elective obstetric or gynecological surgery

[3] Patients willing to provide informed consent

[4] Patients with no signs of active infection

## Exclusion criteria:

[1] Known allergy to antibiotics used in the study

[2] Immunocompromised patients

[3] Patients receiving antibiotics prior to surgery

[4] Emergency surgeries

## Antibiotic regimen:

[1] Group A - Oral Antibiotic Prophylaxis: Patients received oral cephalosporin antibiotic 1 hour before surgery.

[2] Group B - Intravenous Antibiotic Prophylaxis: Patients received intravenous cefuroxime 1.5 g 30 minutes before surgical incision.

## Data collection:

Clinical data collected included:

[1] Age

[2] Body mass index

[3] Type of surgery

[4] Duration of surgery

[5] Post-operative complications

[6] Length of hospital stay

## Outcome measures:

[1] Surgical site infection

[2] Febrile morbidity

[3] Urinary tract infection

[4] Wound infection

[5] Duration of hospital stay

## Statistical analysis:

Data were analyzed using SPSS version 26.0 (IBM Corp., Armonk, NY, USA). Continuous variables were expressed as mean ± standard deviation. Categorical variables were expressed as percentages. Chi-square test and Student's t-test were used. p < 0.05 was considered statistically significant

## Results:

A total of 220 patients were randomized equally to oral (n = 110) or intravenous (IV) (n = 110) antibiotic prophylaxis groups; baseline demographics were similar between groups with a mean age of approximately 34 years and comparable BMI values (Table 1). The case mix was also balanced: cesarean sections accounted for 41.8% of the oral group and 40.0% of the IV group, with hysterectomy and ovarian cystectomy proportions similar between arms (Table 1). Post-operative complication rates did not differ significantly: surgical-site infection occurred in 9 patients (8.2%) in the oral group and 8 patients (7.3%) in the IV group (p = 0.80), febrile morbidity affected 10 (9.1%) and 11 (10.0%) patients respectively (p = 0.82), and urinary tract infection was recorded in 6 (5.5%) versus 7 (6.4%) patients (p = 0.77) (Table 2). More than 90% of patients in both groups experienced normal wound healing (oral 91.8% vs IV 90.9%, p = 0.81), and rates of delayed healing were similar (oral 8.2% vs IV 9.1%, p = 0.82) (Table 3). The mean hospital stay was marginally shorter in the oral group (4.3 ± 1.2 days) than the IV group (4.6 ± 1.4 days), but this difference was not statistically significant (p = 0.19) (Table 3).

## Discussion:

In this study, researchers looked at the effectiveness of oral and intravenous antibiotic prophylaxis for women having gynecological or obstetric procedures. No statistically significant difference in post-operative infection rates was seen between the two groups, according to the data. Results showed that both the oral and intravenous groups were equally effective in preventing surgical site infections (8.2% and 7.3%, respectively). There was no statistically significant difference between the two groups in a randomized clinical study that looked at SSI rates; both the oral prophylactic and intravenous groups measured 14% and 13%, respectively [10]. Another study evaluating antibiotic prophylaxis in gynecological surgery found that adherence to recommended prophylactic guidelines significantly reduced surgical site infections and post-operative complications [3,11]. The comparable outcomes observed in our study may be explained by the ability of oral antibiotics to achieve adequate tissue concentration when administered at an appropriate time before surgery. Furthermore, oral antibiotics provide sufficient coverage against the microorganisms commonly responsible for post-operative infections in gynecological procedures [12]. The present study also found similar rates of febrile morbidity and urinary tract infection between groups. These findings suggest that oral prophylaxis may be as effective as intravenous antibiotics in preventing systemic post-operative infections. An important advantage of oral prophylaxis is its convenience and cost-effectiveness. Oral administration does not require intravenous access or trained personnel and may therefore be particularly beneficial in outpatient surgical settings or in resource-limited healthcare facilities [13]. However, intravenous antibiotic prophylaxis continues to be widely used because it ensures immediate systemic drug levels and is easier to control in terms of timing and dosage. For major surgeries or high-risk patients, intravenous antibiotics may still be preferred. Our findings support the growing body of evidence suggesting that oral antibiotic prophylaxis could be considered a safe and practical alternative to intravenous prophylaxis in selected surgical procedures [14, 15]. Some limitations of the research include a brief follow-up time and a single-center design. These results need to be confirmed by future larger-scale multicenter randomized studies.

## Conclusion:

We show that oral antibiotic prophylaxis is comparable to intravenous prophylaxis in preventing post-operative infections following obstetric and gynecological interventions. Both strategies showed similar rates of surgical site infection, febrile morbidity and post-operative complications. Oral antibiotic prophylaxis offers advantages such as convenience, reduced cost and ease of administration. Therefore, some patients having elective gynecological or obstetric operations may find that oral prophylaxis is more feasible than intravenous antibiotics.

## Figures and Tables

**Table 1:** Demographic characteristics

**Variable**	**Oral Group (n=110)**	**IV Group (n=110)**	**p value**
Mean age (years)	33.8 ±6.5	34.1 ±6.2	0.72
BMI (kg/m^2^)	26.4 ±3.2	26.8 ±3.6	0.48
Cesarean section	46 (41.8%)	44 (40%)	0.79
Hysterectomy	38 (34.5%)	40 (36.3%)	0.78
Ovarian cystectomy	26 (23.6%)	26 (23.6%)	1

**Table 2:** Post-operative complications

**Complication**	**Oral group**	**IV group**	**p value**
Surgical site infection	9 (8.2%)	8 (7.3%)	0.8
Febrile morbidity	10 (9.1%)	11 (10%)	0.82
Urinary tract infection	6 (5.5%)	7 (6.4%)	0.77
No complications	85 (77.3%)	84 (76.4%)	0.88

**Table 3:** Post-operative recovery

**Parameter**	**Oral group**	**IV group**	**p value**
Normal wound healing	101 (91.8%)	100 (90.9%)	0.81
Delayed healing	9 (8.2%)	10 (9.1%)	0.82
Mean hospital stay (days)	4.3 ±1.2	4.6 ±1.4	0.19
